# Structure of a SMG8–SMG9 complex identifies a G-domain heterodimer in the NMD effector proteins

**DOI:** 10.1261/rna.061200.117

**Published:** 2017-07

**Authors:** Liang Li, Mahesh Lingaraju, Claire Basquin, Jérome Basquin, Elena Conti

**Affiliations:** Department of Structural Cell Biology, Max-Planck-Institute of Biochemistry, D-82152 Martinsried, Germany

**Keywords:** NMD, post-transcriptional regulation, *C. elegans*, G domain

## Abstract

Nonsense-mediated mRNA decay (NMD) is a eukaryotic mRNA degradation pathway involved in surveillance and post-transcriptional regulation, and executed by the concerted action of several *trans*-acting factors. The SMG1 kinase is an essential NMD factor in metazoans and is associated with two recently identified and yet poorly characterized proteins, SMG8 and SMG9. We determined the 2.5 Å resolution crystal structure of a SMG8–SMG9 core complex from *C. elegans*. We found that SMG8–SMG9 is a G-domain heterodimer with architectural similarities to the dynamin-like family of GTPases such as Atlastin and GBP1. The SMG8–SMG9 heterodimer forms in the absence of nucleotides, with interactions conserved from worms to humans. Nucleotide binding occurs at the G domain of SMG9 but not of SMG8. Fitting the GDP-bound SMG8–SMG9 structure in EM densities of the human SMG1–SMG8–SMG9 complex raises the possibility that the nucleotide site of SMG9 faces SMG1 and could impact the kinase conformation and/or regulation.

## INTRODUCTION

Nonsense-mediated mRNA decay (NMD) is a eukaryotic surveillance mechanism that degrades aberrant mRNAs containing premature translation termination codons (PTCs) (Popp and Maquat 2013; Lykke-Andersen and Bennett 2014; Karousis et al. 2016). In addition, NMD is a post-transcriptional regulatory mechanism that modulates the expression of physiological mRNAs, affecting the stability of ∼10% of the transcriptome (Lykke-Andersen and Jensen 2015). A universal requirement for NMD is a 5′–3′ RNA unwinding activity that is exerted by the helicase UPF1 and regulated by two associated factors, UPF2 and UPF3. In metazoans, UPF1 is additionally regulated by phosphorylation at the N- and C-terminal regions, a decisive event that creates the binding platform for recruiting SMG6 and SMG5–SMG7, which then target the transcript for degradation (Popp and Maquat 2013; Karousis et al. 2016).

UPF1 phosphorylation is catalyzed by the SMG1 kinase (Yamashita et al. 2001). In human cells, SMG1 copurifies in a complex with SMG8 and SMG9 (Yamashita et al. 2009). Human and nematode SMG8 and SMG9 proteins affect the stability of PTC-containing mRNAs in NMD reporter assays (Yamashita et al. 2009). Consistently, inhibition of human SMG-8 has been shown to ameliorate NMD-exacerbated mutant phenotypes (Usuki et al. 2013). However, general impairment of NMD on natural PTC-containing targets was not detected in *smg-8* mutants in *C. elegans* (Rosains and Mango 2012) and in human subjects carrying homozygous loss-of-function *SMG9* mutations (Shaheen et al. 2016). Human patients with *SMG9* deficiency display widespread transcriptional dysregulation, suggesting a predominant role of SMG9 in post-transcriptional regulation rather than in surveillance (Shaheen et al. 2016).

SMG8 and SMG9 interact with each other and inhibit the kinase activity of SMG1 in vitro (Yamashita et al. 2009; Fernández et al. 2010). Electron microscopy studies have revealed the overall architecture of the SMG1–SMG8–SMG9 complex and the central position of SMG8–SMG9 in this trimeric assembly (Arias-Palomo et al. 2011; Melero et al. 2014; Deniaud et al. 2015). However, the limited resolution of the EM maps and the absence of atomic models have so far hampered a molecular understanding of the mechanisms. In this work, we set out to obtain an atomic model of SMG8–SMG9.

## RESULTS AND DISCUSSION

**FIGURE 1.**
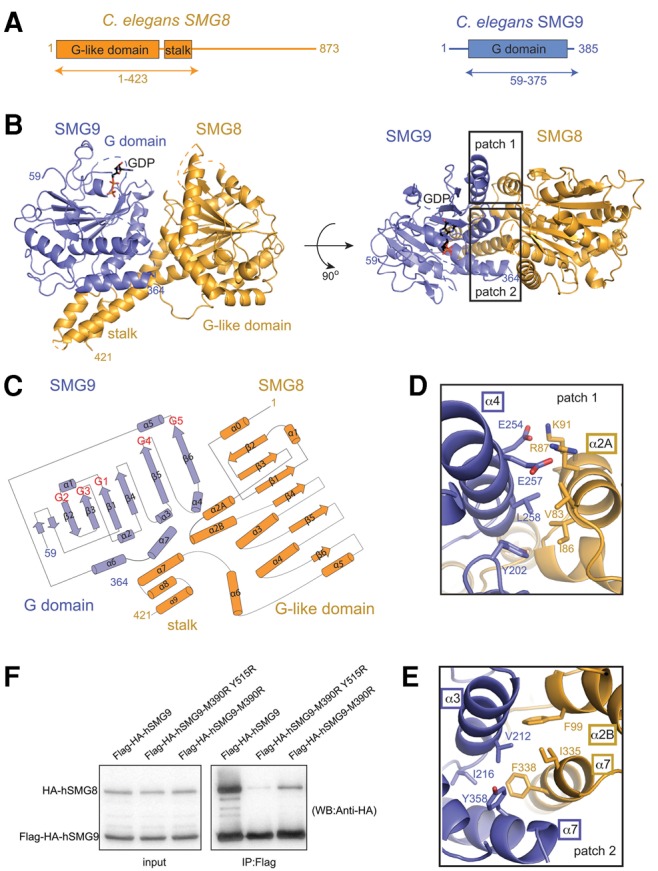
Structure of the conserved core of *C. elegans* SMG8–SMG9. (*A*) Schematic representation of the domain organization of *C. elegans* SMG8 (in orange) and SMG9 (in blue). Domains with a structured fold are shown as rectangles and labeled. Predicted low-complexity regions are shown as lines. The arrows *below* the diagram highlight the parts of the proteins that were crystallized. (*B*) Two views of the crystal structure of the *C. elegans* SMG8c–SMG9c core complex, with the molecules shown in orange and blue, respectively. The two views are related by a 90° clockwise rotation around a *horizontal* axis. The G-like domains and the stalk domain are indicated, as well as the N- and C-terminal residues with ordered electron density. The GDP moiety bound to the SMG9 G domain is shown in stick representation. Disordered loops are highlighted with dotted lines. On the *right*, the two rectangles highlight the two main interaction interfaces (patches 1 and 2) that are shown in more detail *below* in panels *D* and *E*. (*C*) Topological diagram of SMG8c and SMG9c (β-strands shown as arrows and α-helices as cylinders). Loops between secondary SMG9 feature similarities in the so-called G motifs as compared to other G domains. The positions of the G motifs in the loops between secondary structure elements are indicated in red. Note that SMG8c and SMG9c feature additional elements as compared to canonical G domains (α2A, α6 and α6, α7, respectively). (*D*) Zoomed-in view of the interacting residues at patch 1. The molecule is shown after ∼180° rotation around a *horizontal* axis with respect to the view in panel *A*. SMG8c helix α2A and SMG9c helices α3 and α4 are labeled. (*E*) Zoomed-in view of the interacting residues at patch 2. The molecule is shown in a similar orientation as in panel *C*. SMG8c stalk helices α2B and α7 and SMG9c helices α3 and α7 are indicated. (*F*) Coimmunoprecipitation assays of human full-length HA-tagged hSMG8 and Flag-HA-tagged hSMG9 (wild-type or mutants) in transiently transfected HEK293T cells. Cell lysates (input) were immunoprecipitated with Flag binder and detected with an HA-antibody (precipitate) (12% SDS-PAGE gel). The mutated residues in human SMG9 (M390 and Y515) correspond to *C. elegans* SMG9 Leu258 (patch 1, panel *D*) and Tyr358 (patch 2, panel *E*). HA-SMG8 is 111.7 kDa and Flag-HA-SMG9 is 63.6 kDa.

Using bioinformatics analyses and proteolysis experiments, we identified regions *C. elegans* (*C.e*.) full-length SMG8 (873 residues) and SMG9 (385 residues) as sufficient to form a stable heterodimeric core complex (SMG8c, residues 1–423 and SMG9c, residues 59–375, Fig. 1A) and to yield diffracting crystals. After overcoming crystal lattice defects (detailed in Materials and Methods), we solved the structure and refined it at 2.5 Å resolution with *R*_free_ of 26.0% (Table 1).

**TABLE 1.**
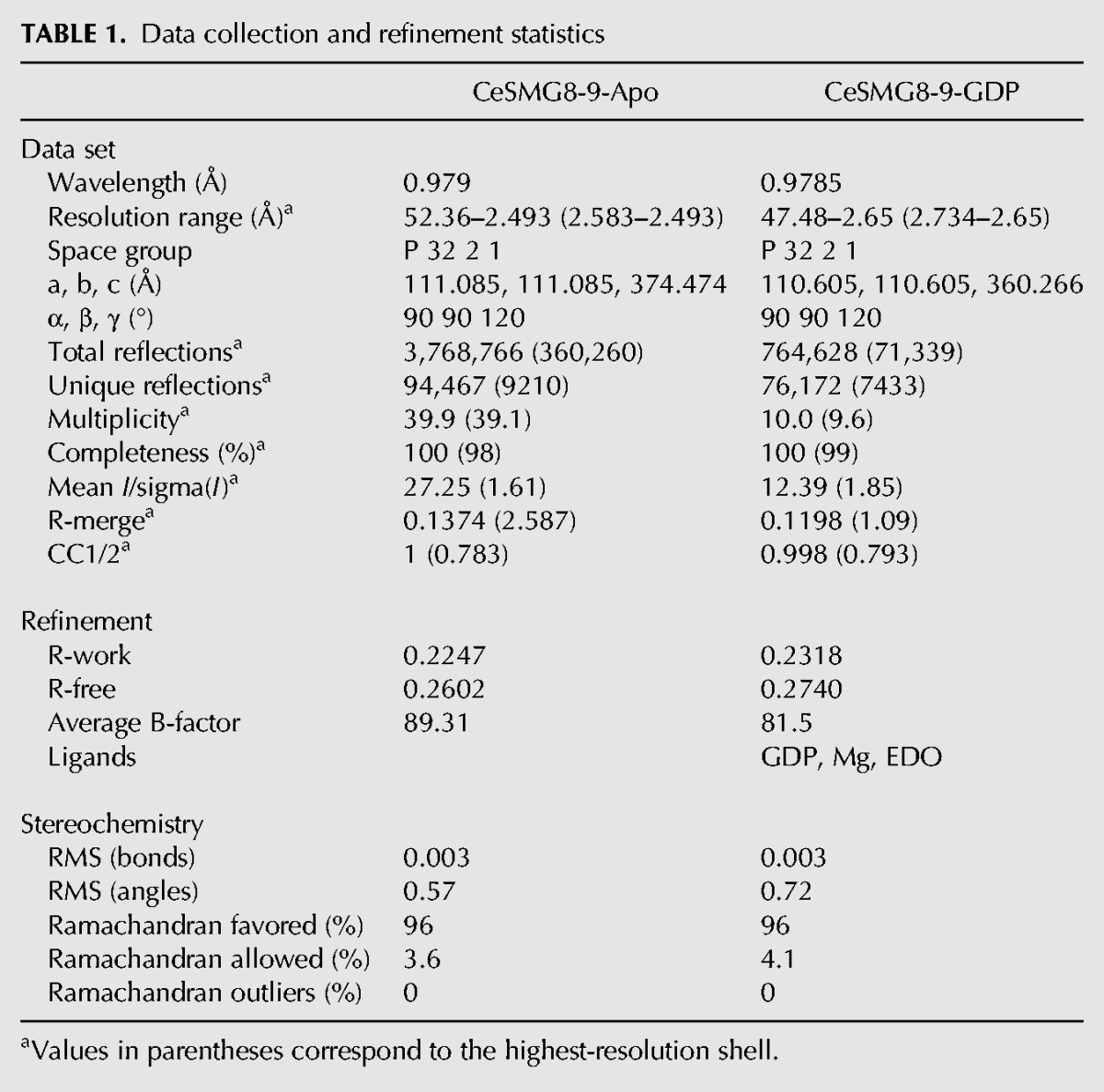
Data collection and refinement statistics

SMG8c and SMG9c contain a similar globular fold with characteristic architecture of G domains along with additional secondary structure elements (Fig. 1B,C). G domains are centered at a mixed β-sheet surrounded by α-helices on the concave and convex surfaces (α1, α5 and α2, α3, α4, respectively) (Wittinghofer and Vetter 2011). The major structural difference between SMG8c and SMG9c is the presence in the former of a helical bundle of three C-terminal helices (α7–α9) that forms a stalk-like protrusion reminiscent of the stalk domain found in GTPases of the dynamin family, such as Atlastin and GBP1 (Fig. 1B; Supplemental Fig. S1; Daumke and Praefcke 2016).

The G domains of SMG8c and SMG9c face each other and interact with part of their convex surfaces (Fig. 1B). In particular, SMG8c helix α2A interacts with SMG9c helices α4 and α3 (patch 1) (in particular Val83^SMG8^, Ile86^SMG8^ with Leu258^SMG9^, Leu261^SMG9^) (Fig. 1D). In addition, the stalk domain of SMG8c folds back on the convex surface of SMG9c (patch 2). Here, SMG8c stalk helices α2B and α7 interact with SMG9c helices α7 and α3 (e.g., Ile335^SMG8^ and Phe338^SMG8^ with Val212^SMG9^ and Tyr358^SMG9^) (Fig. 1E). Many of the hydrophobic interface residues observed in the *C. elegans* SMG8c–SMG9c structure are conserved in the human orthologs (Supplemental Figs. 2, 3), suggesting a similar overall structure. To test this prediction, we engineered mutations in human full-length SMG9 (hSMG9) by substituting Met390 (corresponding to *C. elegans* Leu258^SMG9^) and Tyr515 (corresponding to *C. elegans* Tyr358^SMG9^). We transiently coexpressed full-length HA-tagged hSMG8 and Flag-HA-tagged hSMG9 (wild-type, M390R and M390R, Y515R mutants) in HEK293T cells and carried out coimmunoprecipitation assays with Anti-Flag affinity beads, probing with an anti-HA antibody. We found that the interaction of hSMG8 and hSMG9 observed with the wild-type proteins was indeed strongly impaired by the hSMG9 M390R mutant and almost abolished with the hSMG9 M390R, Y515R double mutant (Fig. 1F).

The relative position of the G-like domains in the SMG8c–SMG9c heterodimer is remarkably similar to that observed in active dimeric GTPases of the dynamin family (Supplemental Fig. S1; Daumke and Praefcke 2016), with the two G domains converging at the loops that are known to harbor the nucleotide-binding motifs (G motifs) in canonical GTPases. However, SMG8 lacks the characteristic residues of G motifs. Another difference is that the single-stalk domain in SMG8c–SMG9c has a different position as compared to the conformations observed in dynamin-like proteins (Supplemental Fig. S1; Byrnes et al. 2013). Finally, the SMG8c–SMG9c heterodimer is formed irrespective of nucleotides, while proteins such as Atlastin or GBP1 dimerize in the presence of GTP analogs (Ghosh et al. 2006; Bian et al. 2011; Byrnes and Sondermann 2011).

**FIGURE 2.**
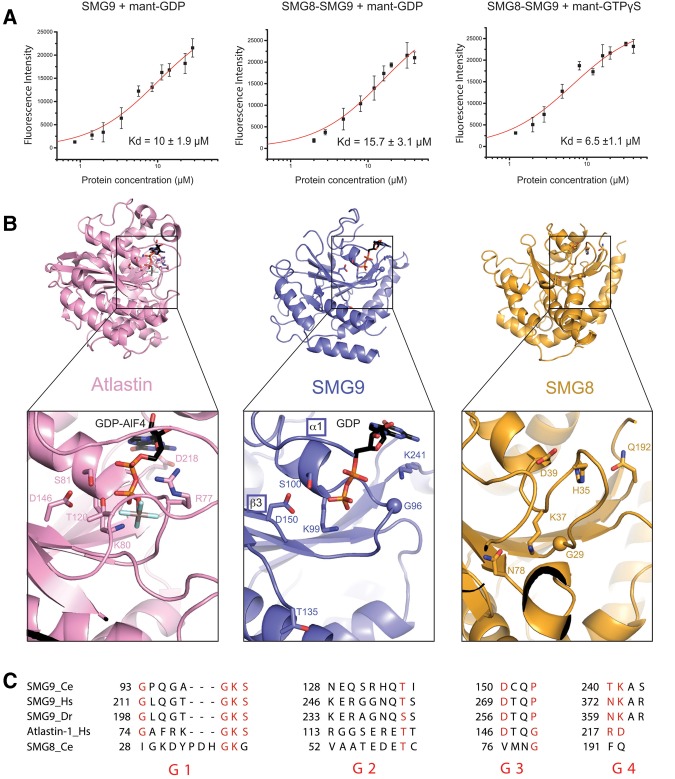
The nucleotide-binding site of SMG9. (*A*) Fluorescence measurements of binding affinities of guanosine-nucleotides to SMG8c–SMG9c and SMG9c using mant-labeled GDP and GTP. The data were fitted to a binding equation describing a single-site binding model to obtain the dissociation constants (*K*_d_). The best fit was plotted as a solid line. The *K*_d_ values and their corresponding errors are the mean and standard deviation of a minimum of three independent experiments. (*B*) Zoomed-in view at the nucleotide-binding site from the structure of SMG8c–SMG9c bound to GDP. The G domain of SMG9 is shown in the same orientation as in Figure 1B, left panel. The G domain of SMG8 and, as comparison, the G domain of Atlastin (bound to the GDP–AlF4 transition-state analog, ref) are shown in a similar orientation after optimal superposition. The nucleotides and important residues at the nucleotide-binding pockets of SMG9 and Atlastin are shown in ball-and-stick representation. Note that Thr135 in GDP-bound SMG9 (*center* panel) corresponds to Thr120 in GDP–AlF4-bound Atlastin (*left* panel). In SMG8, the equivalent site is incompatible with nucleotide binding: His35 and Gln192 would sterically clash with the ribose and base moieties, respectively, and Asp39 would lead to electrostatic repulsion with the phosphates. (*C*) Alignment of the G1–G4 motif sequences of SMG9 from *C. elegans* (Ce), *H. sapiens* (Hs), and *D. rerio* (Dr), and comparison with human Hs Atlastin and Ce SMG8. The position of the G motifs is schematized in Figure 1B: G1 (or P loop) in the β1–α1 loop, G2 (or switch 1) in α1–β2, G3 (or switch 2) in β3–α2, G4 in β5–α4, and G5 in β6–α5. The G5 motif is disordered in the present structure and divergent in sequence and therefore cannot be compared at present.

We tested whether SMG8c–SMG9c can bind guanosine nucleotides. In fluorescence binding assays with mant-nucleotide derivatives, mant-GDP bound SMG8c–SMG9c and SMG9c with a dissociation constant (*K*_d_) of 10 µM and 15 µM, respectively (Fig. 2A). Mant-GTPγS bound SMG8c–SMG9c with a *K*_d_ of 6.5 µM, suggesting a slightly tighter binding in the presence of the nucleotide γ-phosphate (Fig. 2A). In general, the low-micromolar binding affinities we measured for SMG8c–SMG9c are similar to those reported for GBP1 (Praefcke et al. 1999). We proceeded to obtain the structure of a nucleotide-bound SMG8c–SMG9c complex. Although the SMG8c–SMG9c crystals cracked when soaking GTP, GDP soaking experiments were successful. Diffraction data to 2.65 Å resolution (Table 1) showed the presence of well-defined electron density for a GDP moiety in SMG9c but not in SMG8c (Supplemental Fig. S4).

GDP binds SMG9c at a similar position as in Atlastin and GBP1, in particular with similarities at the phosphate-binding loops, e.g., at the motifs G1 (P loop), G2 (switch 1), and G3 (switch 2) (Fig. 2B,C). In SMG9c, the P loop residues Lys99^SMG9^ and Ser100^SMG9^ coordinate the phosphates of GDP. Although parts of the switch regions are disordered in our GDP-bound structure, the switch 2 residue Asp150^SMG9^ is at the position expected for coordinating the magnesium ion, while the switch 1 residue Thr135^SMG9^ is 10 Å away from the position expected upon γ-phosphate binding. There are two notable differences in the G1–G3 motifs of SMG9c as compared to the dynamin-like family. First, there is a conserved proline residue (Pro153^SMG9^, disordered in the present structure) at the position of switch 2 typically occupied by a glycine (Fig. 2B,C). Second, there is a conserved glycine residue (Gly96^SMG9^) in the P loop at the equivalent position of the so-called arginine “finger” (Arg77^Atlastin^) (Fig. 2B,C). Consistent with the absence of such arginine (which stimulates the GTPase activity of dynamin-like proteins in *cis*), we did not detect convincing GTPase hydrolysis in vitro (data not shown). Another significant difference is at the G4 and G5 loops that bind the base of the nucleotide in dynamin-like proteins. The characteristic guanosine specificity determinant of Atlastin and GBP1 in the G4 motif is not present in SMG9 (Fig. 2B,C). At the corresponding position of Asp218^Atlastin^, SMG9 features a conserved lysine residue (Lys241^SMG9^) that stacks with its aliphatic portion on top of the guanine base. With the caveat that motif G5 is largely disordered, none of the interactions in the current structure engage guanine-specific moieties.

**FIGURE 3.**
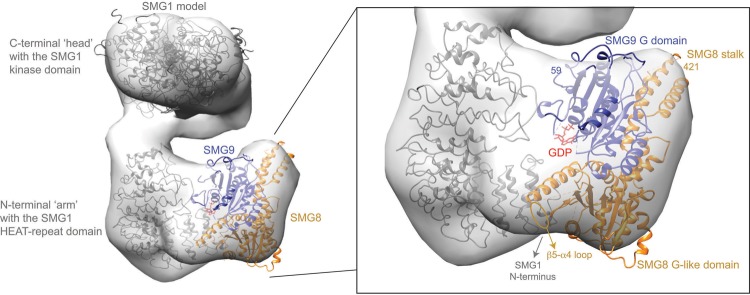
Pseudo-atomic model of a SMG1–SMG8–SMG9 complex. In gray is the EM density of a human SMG1–SMG8–SMG9 complex fitted with a model of human SMG1, as in Deniaud et al. (2015). The remaining density was fitted with the crystal structure of the *C. elegans* SMG8–SMG9 core complex (which lacks the low-complexity SMG9 N-terminal and SMG8 C-terminal regions). In red is the GDP molecule bound to SMG9. The fitting was done in Chimera (Pettersen et al. 2004).

We used our coordinates to progress in the interpretation of cryo-EM structures of human SMG1–SMG8–SMG9 that have been recently resolved at ∼20 Å resolution (Fig. 3; Arias-Palomo et al. 2011; Melero et al. 2014; Deniaud et al. 2015). We fitted a homology model of SMG1 with the kinase domain in the “head” region of the density and the N-terminal HEAT-repeat domain in the “arm” region, as in Deniaud et al. (2015). We positioned the *C. elegans* SMG8c–SMG9c structure in the remaining unoccupied density that is connected to the “arm,” in a density previously shown to correspond to human SMG8–SMG9 (Arias-Palomo et al. 2011). Although the interpretation of low-resolution maps needs to be judged with caution, placing the atomic coordinates appeared to result in a remarkably good fit, whereby the G domain of SMG9 is at the center of the density, with the G-motif loops pointing toward the HEAT repeat region of SMG1 (Fig. 3). In this pseudo-atomic model, SMG8 has a more peripheral position, with the G-like domain approaching the N-terminal end of the “arm” while the stalk is exposed to solvent.

This pseudo-atomic model is generally in agreement with previous biochemical data (Yamashita et al. 2009; Deniaud et al. 2015). The start of the G domain of *C. elegans* SMG9 (residue 59) is near the density of the SMG1 HEAT repeat “arm.” Consistently, the low-complexity N-terminal region of human SMG9 has been shown to interact with the SMG1 HEAT-repeat domain in co-IP assays (Yamashita et al. 2009) and in crosslinking-mass spectrometry experiments (Deniaud et al. 2015). The end of the folded domain of *C. elegans* SMG8 (residue 421) points toward the SMG1 C-terminal “head.” Consistently, the low-complexity C-terminal region of human SMG8 has been shown to contact an insertion domain present in the C-terminal domain of human SMG1 (Deniaud et al. 2015). Finally, the β5–α4 loop of SMG8 faces the density of the SMG1 N-terminal arch. Consistently, the corresponding loop of human SMG8 (residues 290–293) has been shown to contact the SMG1 N terminus in crosslinking-mass spectrometry experiments (Deniaud et al. 2015). Although parts of the SMG9 G motifs as well as the low-complexity regions described above are not present in the current SMG8c–SMG9c crystal structure, the fitting suggests that they might become ordered upon SMG1 binding. In summary, the pseudo-atomic model not only rationalizes how SMG9 recruits the more peripheral SMG8 to the SMG1 complex (Deniaud et al. 2015), but also has predictive value because it raises the hypothesis that the nucleotide-binding state of SMG9 might impact on the entire complex.

## MATERIALS AND METHODS

### Protein expression and purification

We analyzed the amino acid sequence of SGM8 and SMG9 proteins from different species in an effort to identify orthologs that would be best suited for crystallization. We selected the *C. elegans* (*C.e.*) proteins since they are 10%–25% smaller and therefore likely more compact than their human counterparts. *C.e.* SMG8 (873 residues) and SMG9 (385 residues) were subcloned from a *C.e.* cDNA library with standard PCR protocols in a single MultiBac expression vector (pFL) (Fitzgerald et al. 2006). SMG8 was cloned into the multiple cloning site 1 (MCS1) of the pFL vector using Xma1 and Nhe1, while SMG9 was cloned into the multiple cloning site 2 (MCS2) using BamHI and SalI. Coexpression was crucial to obtain the heterodimer: Although SMG9 could be expressed and purified in a soluble form, SMG8 was insoluble when in isolation (data not shown). Rounds of limited proteolysis and optimization of the expression constructs narrowed down the SMG8c–SMG9c core complex (*C.e*. SMG8 1–423 and SMG9 59–375). SMG8c–SMG9c were coexpressed in baculovirus-infected Hi-Five insect cells (Invitrogen) at 26°C for 70 h. Cells were lysed in 25 mM Tris pH 8.0 with 300 mM NaCl, and 20 mM imidazole was supplemented to the supernatant before loading onto the nickel column. The complex was purified by nickel-based affinity chromatography via a C-terminal hexa-histidine tag on *C.e*. SMG8, and subsequent ion exchange (Heparin HiTrap) and gel-filtration chromatography (Superdex200, equilibrated with 25 mM Tris, 300 mM NaCl, pH 8.0). SelenoMethionine (SeMet) substituted proteins were expressed in insect cells with similar protocols that we reported previously (Halbach et al. 2013). The purification procedure of the SeMet-substituted complex was the same as for the native protein, except that all buffers were degassed and 4 mM β-mercaptoethanol and 2 mM DTT were added before and after elution from the Ni^2+^–NTA resin, respectively. Mass spectrometry analysis showed the presence of ∼60% SeMet incorporation in the purified complex.

### Crystallization and structure determination

*C.e.* SMG8c–CeSMG9c crystallized by vapor diffusion in several PEG conditions at pH 8.0 and 10°C. These initial crystals diffracted to ∼3.0 Å resolution and could be processed in a hexagonal spacegroup, but analysis of the cumulative intensity distribution showed the presence of merohedral twinning with a twin fraction close to 0.5. Additive screening allowed us to identify yttrium chloride as an effective chemical compound to overcome the twinning problem. The best un-twinned crystals were grown by hanging-drop vapor diffusion in drops formed by equal volumes (1.5 µL) of protein (6.8 mg/mL in gel filtration buffer supplemented with 0.11 mM YCl_3_) and crystallization buffer (10% PEG3350, 0.1M Tris pH 8.5). SeMet crystals were obtained using the same conditions, but adding tris(2-carboxyethyl)phosphine (TCEP, to limit SeMet oxidation) and covering the reservoir buffer with paraffin oil (to slow drop evaporation and increase crystal size). All crystals were cryoprotected with the crystallization buffer supplemented with 25% ethylene glycol prior to cryo-cooling and data collection.

Diffraction data were collected at 100K at the Swiss Light Source (SLS) beamline PXII. Diffraction data were collected at the selenium K-edge peak wavelength and were processed with XDS (Kabsch 2010). The crystals belong to a trigonal P3221 space group with three copies of the complex in the asymmetric unit related by noncrystallographic symmetry. We used SHELX for phasing (Sheldrick 2010) and phenix.autobuild for initial model building (Adams et al. 2010). We completed the model with iterative rounds of manual building in Coot and refinement with phenix.refine. The three independent copies of the complex in the asymmetric unit are very similar and contain most of the polypeptide chains, except disordered loop regions. The copy of SMG8c–SMG9c described in the text contains SMG8 residues 1–416 (with the exception of disordered loops between residues 193–211, 256–288, and 356–386) and SMG9 residues 59–363 (with the exception of disordered loops between residues 124–134, 152–172, and 284–311) (Table 1).

Native crystals were soaked with 10 mM GDP for 5 min prior to freezing. The structure of *C.e.* SMG8c–SMG9c–GDP was determined by molecular replacement with Phaser using the SeMet-derivatized CeSMG8–9 structure as a search model. The model was completed with Coot (Emsley et al. 2010) and refined with phenix.refine (Adams et al. 2010).

### Nucleotide-binding experiments

The affinities for GDP were determined by fluorescence measurements on an Infinite M1000 Pro (Tecan). Experiments were carried out at 21°C in a buffer containing 25 mM Tris pH 8.5, 150 mM NaCl, and 5 mM MgCl_2_. Increasing protein concentrations were incubated with 1.67 µM of methylanthraniloyl (mant) labeled GDP for 30 min at room temperature. The experiments were carried out with the fragments crystallized, since the full-length proteins were prone to degradation of the low complexity sequences. Fluorescence of mant-GDP was excited at 355 nm and emission spectra were then monitored from 400 to 500 nm, with emission maxima detected at 448 nm. The intrinsic protein fluorescence as well as the mant-nucleotide background was subtracted from the curves. Curve fittings were done with Origin with a one-to-one binding model and are consistent with the presence of one molecule of nucleotide per heterodimer. Curves were done in triplicate. Similar approaches were used to determine the binding affinities for GTPγS.

### Coimmunoprecipitation assays

Both the SMG8 and SMG9 were cloned in a vector containing the EF-1 α promoter and with an N-terminal Flag tag and N-terminal HA tag using EcoRI and NotI restriction sites. HEK293T cells were cultured in Dulbecco's modified Eagle medium containing 10% fetal bovine serum (Gibco), 100 U/mL penicillin, and 0.1 mg/mL streptomycin (Gibco) at 32°C/5% CO_2_. Plasmids were transfected with polyethyleneimine (Polysciences Inc., 1 mg/mL) for protein interaction studies. HEK293T cells were collected from confluent six-well plates after 72 h of transient transfection. Cells were lysed in 0.5 mL of lysis buffer containing 50 mM Tris, pH 7.4, 150 mM NaCl, supplemented with protease inhibitor cocktail (Roche) and DNase I. The lysate was centrifuged at 16,000*g* for 30 min at 4°C. Twelve microliters of Anti-Flag M2 sepharose beads (Sigma) were added to supernatant for 1 h at 4°C. Beads were washed four times with 1 mL of buffer containing 50 mM Tris, pH 7.4, 300 mM NaCl, and proteins were eluted with 25 µL of lysis buffer supplemented with 100 µg/mL flag peptide (Sigma-Aldrich, F3290). Eluted proteins were run on 12% polyacrylamide gels and transferred onto polyvinylidene difluoride membrane (0.45 µm pore size) (Millipore Immobilon-P) for Western blotting. Anti-HA (Covance, MMS-101 R) antibody and horseradish peroxidase–coupled goat anti-mouse (Millipore, AQ502A) secondary antibody were used in combination with ECL prime Western blotting detection reagent (GE healthcare) for detection of Flag-HA and HA-tagged proteins via Western blotting.

## DATA DEPOSITION

The coordinates have been deposited in the Protein Data Bank with accession codes 5NKM (SMG8-SMG9) and 5NKK (SMG8-SMG9-GDP).

## SUPPLEMENTAL MATERIAL

Supplemental material is available for this article.

## Supplementary Material

Supplemental Material
