# Supplementary material for: Structure of a SMG8–SMG9 complex identifies a G-domain heterodimer in the NMD effector proteins
Source: RNA. 2017 Jul;23(7):1028–34. doi: 10.1261/rna.061200.117 (PMC5473137; doi:10.1261/rna.061200.117)
Supplement: Supplemental Material [file supp_23_7_1028__index.html]

Structure of a SMG8–SMG9 complex identifies a G-domain heterodimer in the NMD effector proteins — Supplemental Material 

# Structure of a SMG8–SMG9 complex identifies a G-domain heterodimer in the NMD effector proteins

## Supplemental Material

Supplemental Material

- Supplemental\_Figure\_1.jpg
- Supplemental\_Figure\_2a.jpg
- Supplemental\_Figure\_2b.jpg
- Supplemental\_Figure\_3.jpg
- Supplemental\_Figure\_4.jpg
