# Supplementary figures and images for: Structure of a SMG8–SMG9 complex identifies a G-domain heterodimer in the NMD effector proteins
Source: RNA. 2017 Jul;23(7):1028–34. doi: 10.1261/rna.061200.117 (PMC5473137; doi:10.1261/rna.061200.117)

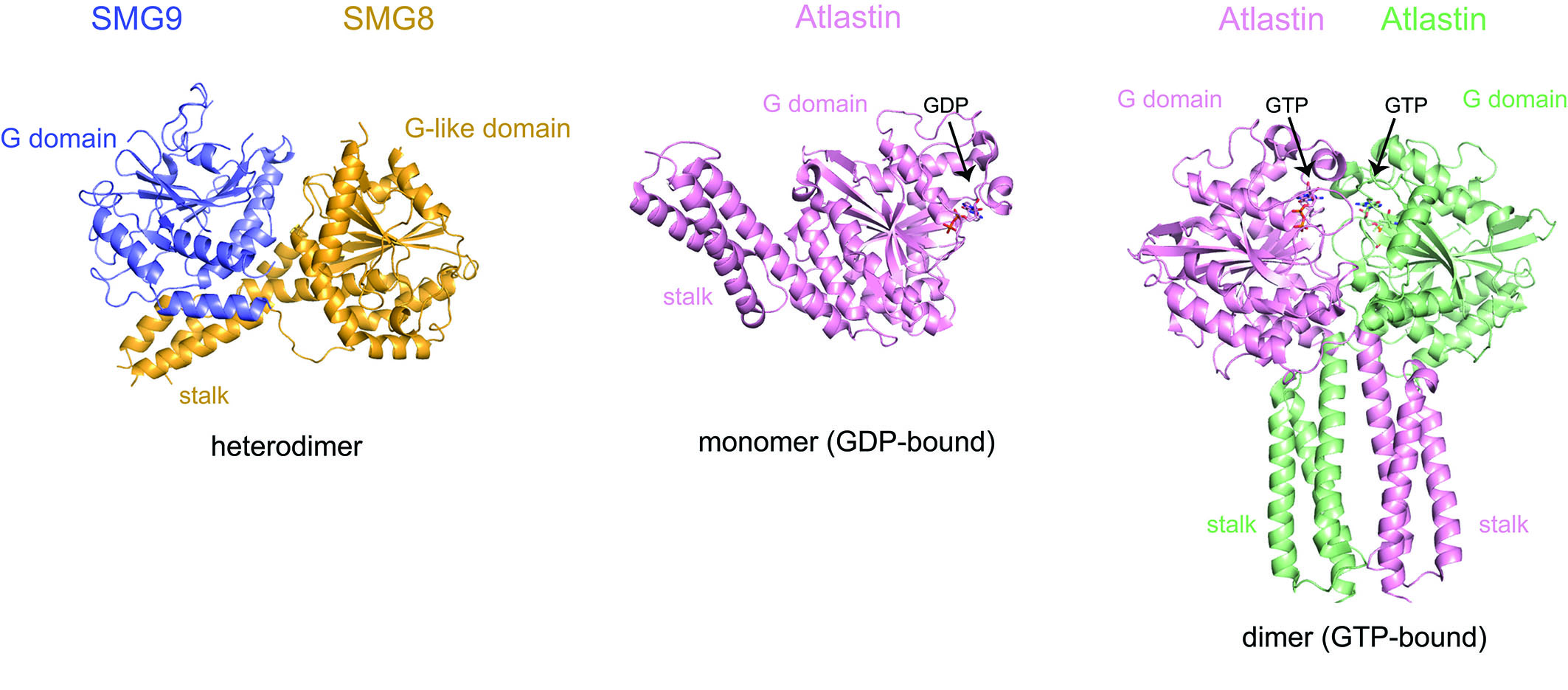

Supplement: Supplemental Material [file supp_061200.117_Supplemental_Figure_1.jpg]

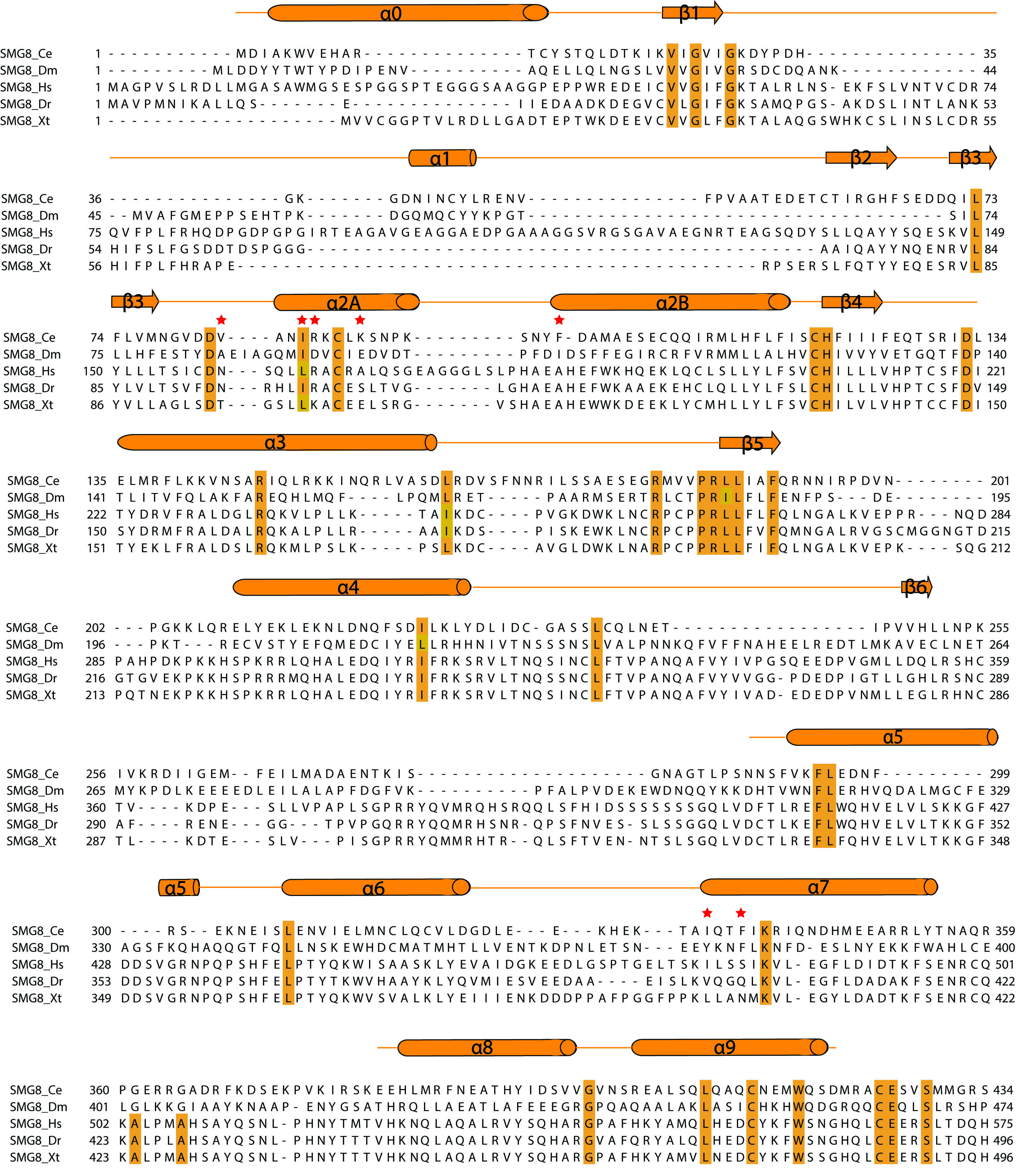

Supplement: Supplemental Material [file supp_061200.117_Supplemental_Figure_2a.jpg]

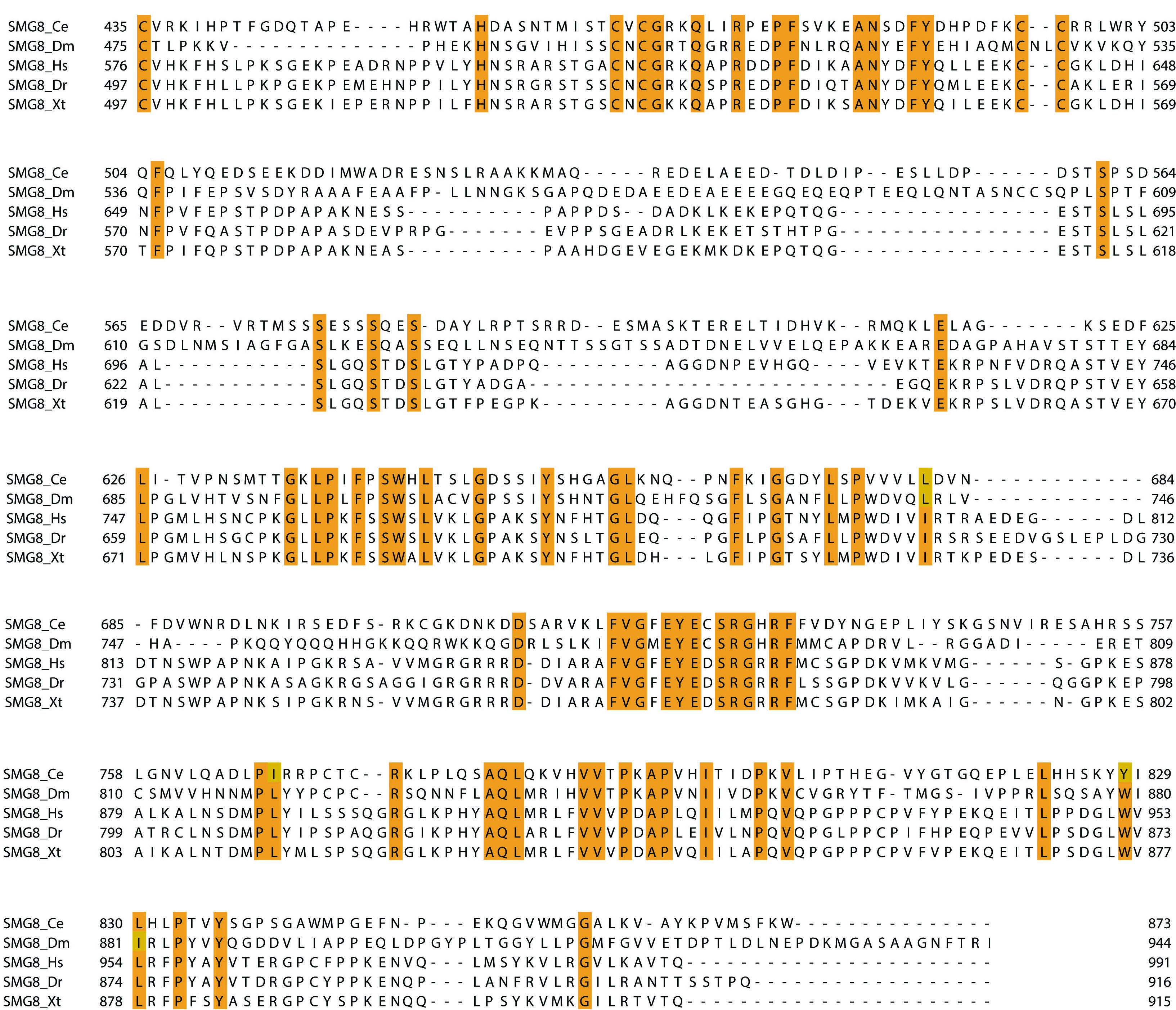

Supplement: Supplemental Material [file supp_061200.117_Supplemental_Figure_2b.jpg]

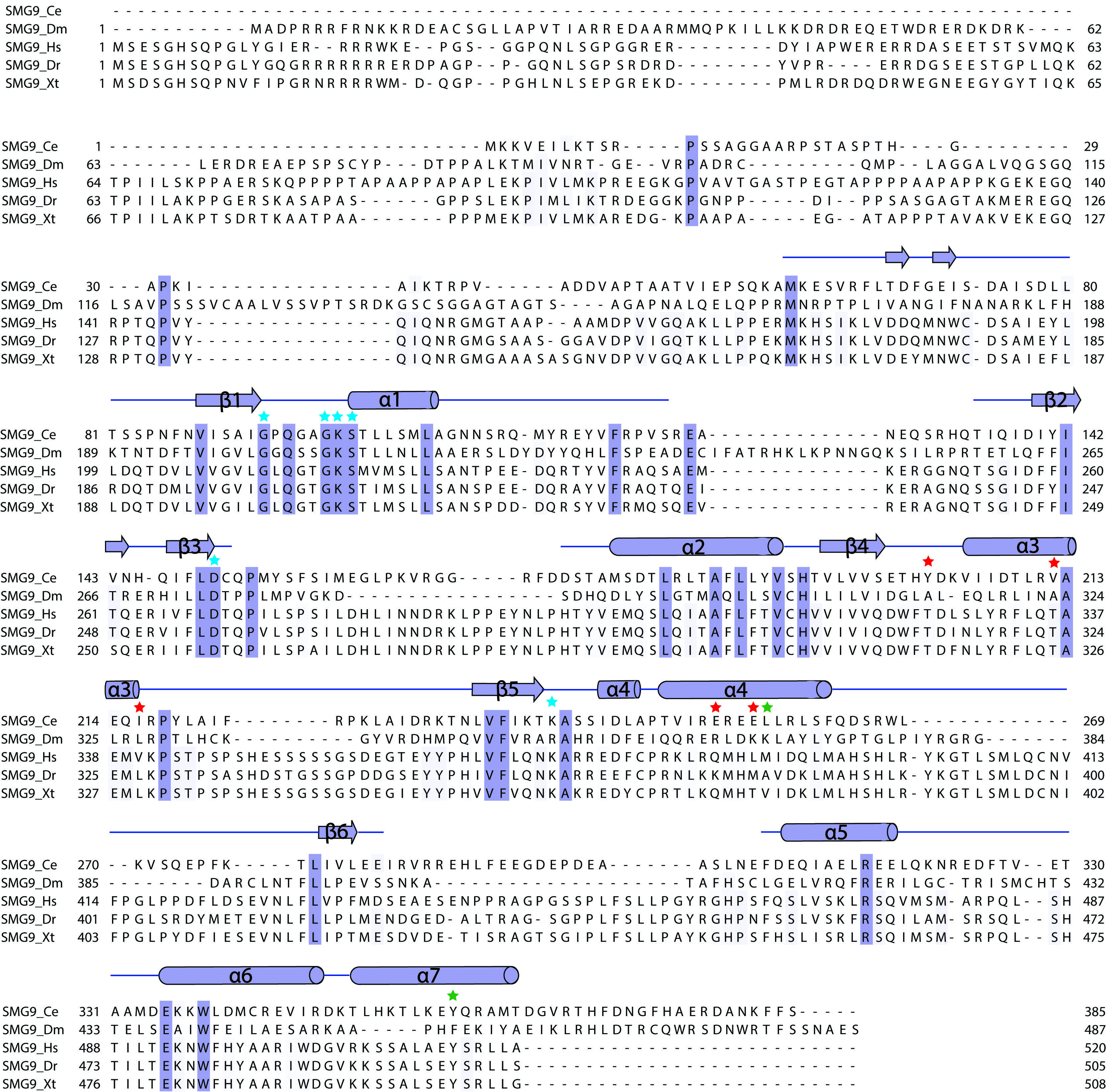

Supplement: Supplemental Material [file supp_061200.117_Supplemental_Figure_3.jpg]

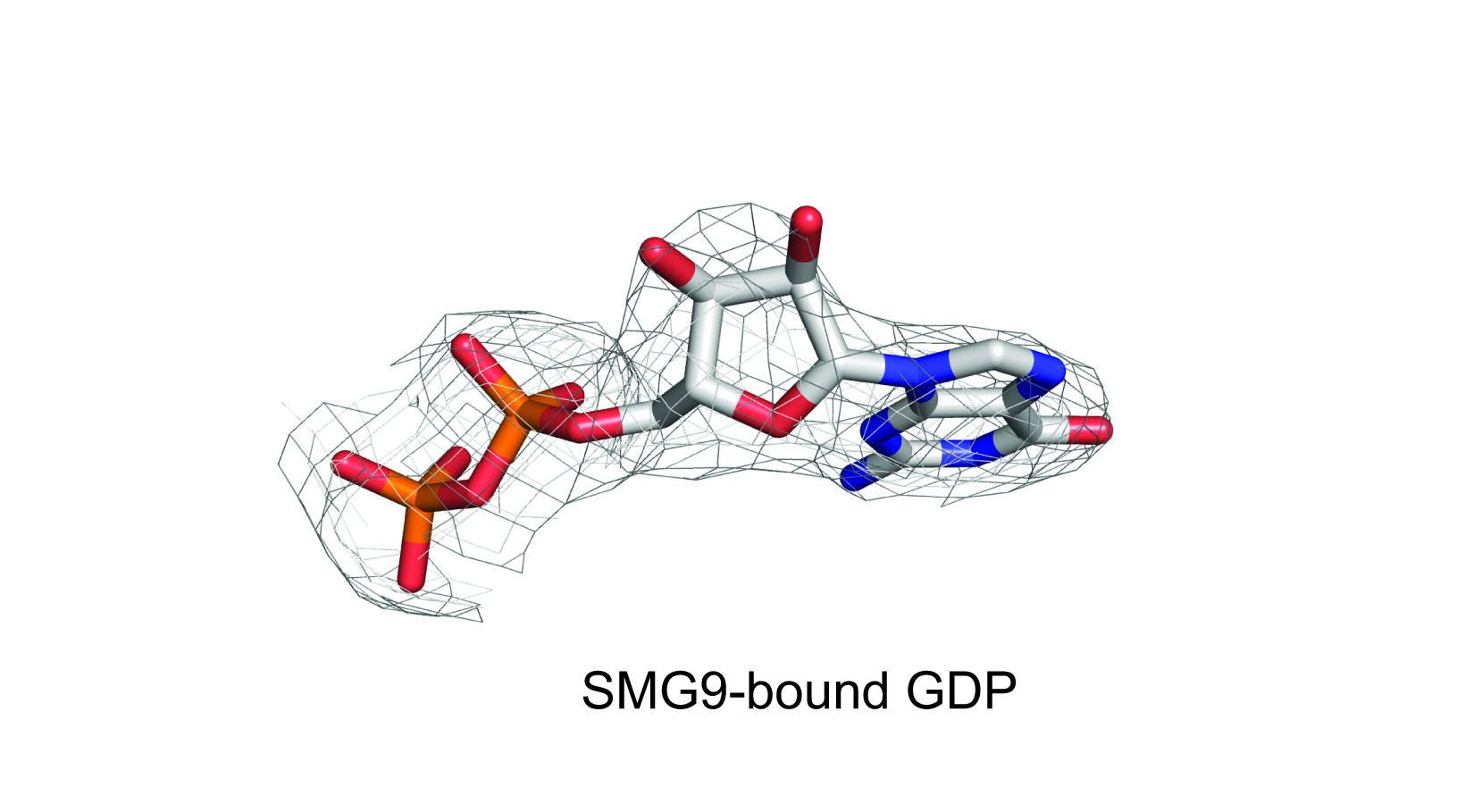

Supplement: Supplemental Material [file supp_061200.117_Supplemental_Figure_4.jpg]
